# Incidental findings in population imaging revisited

**DOI:** 10.1007/s10654-016-0123-0

**Published:** 2016-02-09

**Authors:** Eline M. Bunnik, Meike W. Vernooij

**Affiliations:** Department of Medical Ethics and Philosophy of Medicine, Erasmus MC, Wytemaweg 80, 3015 CN Rotterdam, The Netherlands; Departments of Radiology and Epidemiology, Erasmus MC, Wytemaweg 80, 3015 CN Rotterdam, The Netherlands

Imaging techniques are deployed in human subjects research on an increasingly large scale. Worldwide, images of the brain, the abdomen or the whole body are acquired in clinical and population-based cohort studies, and in neuroscience, cognitive science and behavioural science studies. Many of these imaging studies are performed in volunteers who are presumed healthy and free of any symptoms. Yet, even in healthy volunteers, structural abnormalities are detected quite frequently, in approximately 2–3 % of MRI scans of the brain [1, 2], and possibly in over a third of whole-body MRI scans [3]. So-called incidental findings may be of clinical or reproductive significance to research participants. Incidental findings have commonly been regarded as findings that are unrelated to the aims of the study and are discovered unexpectedly in the course of conducting research [4].

The word ‘incidental’ literally means “being likely to ensue as a chance or minor consequence” or “occurring merely by chance or without intention or calculation” [5]. Incidental findings have historically been understood as observations ‘stumbled upon’ by researchers or radiologists. This presupposes a passive, unprepared observer, upon whom an abnormality ‘happens’ by chance. The ethics of incidental findings has generally aligned with this notion. Ethical guidance on the detection, management and communication of incidental findings requires researchers or institutions to have (no more than) a ‘contingency plan’ in place: *if* researchers stumble upon something that may be relevant to the health of the participant, *then* they should act. Most guidance documents do state that the actions to be taken (e.g. consultation of an expert radiologist for confirmation of the finding, communication of the finding to the participant or their physician) should be outlined in a predesigned protocol or pathway to be approved by an institutional review board [4, 6, 7], which should be communicated beforehand with research participants. Thus, current ethical guidance covers mostly what happens *after* incidental findings have been detected, but it does not expressly address *whether* incidental findings should be detected or what actions should be taken to avoid or ensure the detection incidental findings. A prominent topic of debate is whether routine review of research scans is required. Practices vary considerably across countries and studies [7, 8]: among the largest population-based studies currently being conducted in Europe, approaches for the detection of incidental findings range from only limited handling of those findings that are stumbled upon by radiographers during scan acquisition [9] to having all scans read by dedicated radiologists [10]. In what follows we explain why the research community must take a proactive role and resolve this debate.

## Incidental findings are no longer unexpected

As imaging of healthy participants is becoming more widespread and researchers’ experience with the detection, management and clinical follow-up of incidental findings is consequently becoming more thorough, incidental findings can no longer be considered to be unexpected. The prevalence of clinically significant incidental findings among healthy participants is known to be around 2.7 % in MRI of the brain [1], which implies a number needed to scan of around 37 for one significant finding [1, 2]. The prevalence may be slightly higher in elderly subjects [2], but even in children and adolescents, incidental findings occur [11]. Incidental findings may be (much) more frequent in other areas of the body, such as the abdomen or chest [3, 12]. Based on this accumulating evidence, researchers can thus anticipate, firstly: that incidental findings will occur; and, secondly, for specified study populations (i.e. depending on age range, health status): how many and what types of incidental findings are likely to occur.

## Incidental findings are no longer accidental

Incidental findings are the result not of chance, but of choices made—intentionally or unintentionally—by the researcher, research team and/or institution. The technical parameters of the imaging study, the training and instruction of the research staff, the time and resources spent on the reviewing of scans, are all factors that will affect the detection of incidental findings.

First, researchers can influence the occurrence of incidental findings through the design of the scan protocol itself, through decisions regarding the technical parameters or the sequences to be acquired and the quality thereof: e.g. in brain imaging, T2 weighted and fluid-attenuated inversion recovery (FLAIR) images are of greater diagnostic utility than T1 weighted images or functional MRI and will generally lead to the detection of more incidental findings [2, 13].

Second, whether or not incidental findings are detected depends in part on the person who is looking at the scans. As radiographers are trained differently from radiologists, they will perceive different things when looking at the same scan. Likewise, PhD-researchers in neuroscience or psychology university departments will perceive differently from hospital-based research staff, and so on. Not only the researchers’ background and training, but also their personality traits (e.g. conscientiousness) or state of mind (e.g. how much sleep they have had) may affect what they see when looking at MRI scans.

Third, whether incidental findings are detected depends on the instructions given to the person who is looking at the scans: are they asked to only check the quality of the images or also to check for abnormalities? Are they *trained* to review scans for abnormalities? And even: how much importance is placed on the task of reviewing by supervisors? Does the reviewer have access to expert opinion when in doubt?

Fourth, what the researcher sees on MRI scans depends on the time and the timing allocated for (clinical) review of scans. Are scans reviewed on the spot or later on? Are they to be glanced over in a few seconds or scrutinized for minutes? Are those who are asked to review scans allotted sufficient time or are they working under stress?

Additional factors that can influence the detection of incidental findings may include the goals of the research (medical or non-medical), the location of the research centre (whether the research setting is hospital-based or, for instance, a neuroscience lab at a psychology department), and the research population (e.g. healthy student-volunteers or the elderly). The same researcher may see differently when reviewing scans for a medically oriented study or a study with a different (e.g. behavioural) orientation, in a hospital-based research setting or in a neuroscience lab. Context often colours perception.

These and others factors are likely to influence the way scans are ‘looked at’. Technical and organizational factors work together to fashion the salience of abnormalities and therewith to bring about the detection of incidental findings. The occurrence of incidental findings is thus dependent not only on the age and health status of research participants, but also—and potentially equally or more so—on choices made by researchers concerning the scan protocol, the selection and instruction of scanner operators, and the clinical review (or not) of scans. Incidental findings are not ‘discovered’ but ‘created’. They are not accidental: knowingly or unknowingly, they are orchestrated.

## Ethical implications

Although incidental findings do not always lead to health benefits [14] and are associated with risks (e.g. medical costs, psychological harms and burdens of unnecessary follow-up testing and overtreatment) [13, 15], it is widely endorsed that *if* a researcher detects a finding of clinical relevance, he or she has a ‘duty to disclose’ the finding to the participant [6, 7, 16]. We have tried to unravel the black box of the ‘if’ within that maxim: incidental findings do not happen accidentally, but are brought about. They are the result of technical, social and institutional factors that are controlled by researchers, research teams, institutional review boards and funding organizations. The research community should take responsibility for the—controllable—factors within the research setting that will affect the likelihood of detection of incidental findings.

Now what does taking responsibility mean? What choices—regarding the scan protocol, the staff tasked with reviewing scans and their training and instruction—should be made? Are all choices equally justifiable? Given the variation in research settings, research aims and professionals involved in research imaging, it will hardly be possible to formulate a one-size-fits-all recommendation for the management of incidental findings. Rather, ethical guidance should be differentiated across research settings. We will take routine review of research scans as an example of how to go about the task of differentiation.

The crucial consideration in the ethics of incidental findings, we argue, is this: participants’ expectations should be met—to a reasonable extent. Research participants tend to trust that abnormalities—if present—will be detected and communicated to them [15]. A mismatch between practices of incidental findings and subjects’ expectations may undermine informed consent and may lead to harm (e.g. through false reassurance) [17, 18]. Mismatches can basically be resolved in two ways: first, by ‘lifting up’ research practice (e.g. by indeed offering routine review); or second, by bringing down participants’ expectations (e.g. by informing participants about a no-feedback policy). The latter may be morally unacceptable in some—but not all—research settings.

Routine clinical review is time-consuming and costly, and may not be feasible for all research groups or institutions. Some groups may thus decide to instruct radiographers to review scans on the spot for quality only, and to avoid the detection of abnormalities. This approach is often accompanied by an informed consent process in which participants are told that ‘no one will look at the scans’ and/or that ‘no feedback will be given’. This policy may be defensible in certain research contexts, for instance, in neuroscience, cognitive science or behavioural science laboratories in which studies are conducted, for example, using fMRI of the brain among healthy student–volunteers. It is conceivable that in such research contexts, the informed consent process can be successfully used to downplay participants’ expectations [19] and to rebut the so-called diagnostic misconception among participants [16]. After all, it can be explained that the images acquired are of limited diagnostic utility: potentially significant abnormalities can easily be missed (i.e. false negative errors), even by trained radiologists, while visible abnormalities may be artefacts (i.e. false positive errors) [16]. It would be a waste of resources to have qualified radiologists review fMR images of the brain acquired for non-medical research purposes: fMR images lack informative value.

In other research contexts, however, we contend that—some form of—routine review of research scans is morally required. For instance, research teams that are hospital-based, have access to clinical expertise and/or conduct health-related studies for which sequences of diagnostic utility are acquired, such as T1 and T2 weighted and/or FLAIR images, should perform some sort of structured review for clinically relevant abnormalities. For in such clearly *medical* research settings, research participants will—reasonably—expect obvious and important abnormalities to be detected and fed back [15]. Furthermore, the vast majority of participants of clinical or population-based cohort studies *prefer* to know about incidental findings, and indicate an interest in learning relevant health information to which they might otherwise not have access [18, 20]. The principle of reciprocity [21] coupled with the—logistically—relative ease with which diagnostic-quality scans can be checked for apparent abnormalities by trained personnel, requires researchers to arrange routine review. Though routine review by a clinical radiologist need not entail unbearable financial costs [22], less costly pathways may be feasible, too: research personnel can be trained to distinguish normal from abnormal scans and to send the latter on—in a second step–for expert opinion. An expert radiologist should confirm the clinical significance of an apparent abnormality before it is fed back to the research participant [6, 7]. Therefore, researchers should make arrangements for second-step consultation with expert radiologists prior to the start of any imaging study. A two-step approach may be more feasible in some body parts than others, e.g. brain imaging versus abdominal imaging. Any selected approach for the handling of incidental findings should be implemented in a standardized manner, communicated with research participants as part of the informed consent process, and monitored through a quality assurance program.

Routine review of research scans is but one controllable factor in the ‘creation’ of incidental findings. It is beyond the scope of this article to outline differentiated ethical recommendations for all controllable factors in all research settings.

## Conclusion

In the above, we have tried to show that it will no longer suffice to have established *a* pathway for *when* incidental findings have ‘happened’. Researchers and research institutions must make up their minds: should they facilitate or avoid the detection of incidental findings? We have argued that there is indeed an answer to that question, though it depends on the research setting. Researchers should plan ahead and assume their decisive roles in the orchestration of incidental findings. Ethically responsible pathways for the detection, management and communication of incidental findings should accommodate research participants’ expectations. In population-based cohort studies of a clearly medical character or studies in which diagnostic-quality images are acquired, all research scans should be reviewed—in one way or another—for relevant abnormalities.
